# Chemically Induced Resistance to Pathogen Infection in *Arabidopsis* by Cytokinin (*Trans*‐Zeatin) and an Aromatic Cytokinin Arabinoside

**DOI:** 10.1111/mpp.70200

**Published:** 2026-01-09

**Authors:** Martin Hönig, Anne Cortleven, Ivan Petřík, Radim Simerský, Magdalena Bryksová, Ondřej Plíhal, Thomas Schmülling

**Affiliations:** ^1^ Institute of Biology/Applied Genetics, Dahlem Centre of Plant Sciences Freie Universität Berlin Berlin Germany; ^2^ Department of Chemical Biology, Faculty of Science Palacký University Olomouc Czech Republic; ^3^ Laboratory of Growth Regulators, Faculty of Science, Palacký University & Institute of Experimental Botany The Czech Academy of Sciences Olomouc Czech Republic

**Keywords:** *Arabidopsis thaliana*, biotic stress, chemically induced resistance, cytokinin, cytokinin arabinoside, pathogen attack

## Abstract

This study compares the ability of the cytokinin (CK) *trans*‐zeatin (*t*Z) and the CK sugar conjugate 6‐(3‐methoxybenzylamino)purine‐9‐arabinoside (BAPA) to induce resistance against the bacterial pathogen *Pseudomonas syringae* in *Arabidopsis thaliana* . Treatment with either *t*Z or BAPA significantly reduced bacterial growth after a later infection. This chemically induced resistance (IR) required the CK receptor AHK3, highlighting its critical role in mediating resistance by *t*Z and BAPA. This is remarkable as these compounds show either high or no affinity for this CK receptor, respectively. Surprisingly, *t*Z, but not BAPA, induced the expression of CK response genes, including *ARR5*, suggesting divergent mechanisms of action. Resistance caused by both compounds was abolished in the *npr1* mutant, underpinning the functional relevance of the salicylic acid (SA) signalling pathway. Transcriptomic analysis showed that both BAPA and *t*Z triggered the expression of distinct sets of genes associated with SA and reactive oxygen species (ROS) but not with jasmonic acid (JA) signalling. BAPA and, to a lesser extent, also *t*Z activated pattern‐triggered immunity (PTI) signalling genes, including genes responsible for PTI signal amplification (*PREPIP2*) and pathogen‐associated molecular pattern (PAMP) signalling (*PH1*, *IDL6*). This supported the hypothesis that the PTI pathway mediates the protective effect. Similarities and differences of chemically triggered IR by *t*Z and BAPA, as well as their potential for application, are discussed.

## Graphical abstract

*Trans*‐zeatin (tZ) and the cytokinin conjugate BAPA induce plant resistance to *Pseudomonas syringae* . Both require the AHK3 and NPR1 receptors, but BAPA lacks unfavourable cytokinin activities, indicating its potential for application.

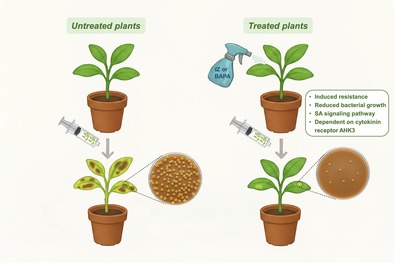

## 1 Introduction

To defend against pathogens, plants have evolved a variety of detection and response systems, including a two‐layered inherent immune system composed of pattern‐triggered immunity (PTI) and effector‐triggered immunity (ETI) (Pruitt et al. 2021). Additionally, plants are capable of enhancing their basic resistance through a process known as induced resistance (IR) when triggered by factors such as pathogens, herbivores or chemicals (Bagheri and Fathipour 2021; Hönig et al. 2023). This response reduces their vulnerability to future threats. IR can manifest locally, affecting only the specific areas exposed to the triggering factor, or systemically (ISR), enhancing resistance throughout the entire plant (De Kesel et al. 2021). Upon contact with the IR stimulus, plants can directly induce plant defence mechanisms or they can also remember the stress stimuli and prepare themselves for enhanced resistance against future stresses. Such plants are then called ‘primed’ because they can respond earlier, faster and/or stronger to a following stress (Hilker and Schmülling 2019; Mauch‐Mani et al. 2017). All IR phenotypes can be considered the result of a combination of directly induced defence responses, primed defence responses, local resistance and systemic resistance in varying proportions (De Kesel et al. 2021). A variety of naturally occurring and synthetic compounds have been reported to cause IR in plants, many of which achieve this primarily through a process known as chemical priming (Hönig et al. 2023). The phytohormone cytokinin (CK), which is best known for its ability to regulate plant growth and development (Kieber and Schaller 2018), and also participates in regulating responses to biotic and abiotic stresses (Cortleven et al. 2019), has been shown to induce resistance against a broad spectrum of biotrophic and hemibiotrophic pathogens (Albrecht and Argueso 2017).

In *Arabidopsis*, Choi et al. (2010) revealed that CK modulates salicylic acid (SA) signalling to enhance resistance against *Pseudomonas syringae* pv. *tomato* DC3000 (Pst). The CK‐activated transcription factor ARR2 was shown to bind to the SA response factor TGA3 and activate the expression of the pathogenesis response gene *PR1* and the SA biosynthesis gene isochorismate synthase 1 (*ICS1*). The binding of ARR2 to TGA3 was dependent on NPR1, a key regulator of the SA signalling pathway.

Choi et al. (2010) further demonstrated that pretreatment with *trans*‐zeatin (*t*Z) for 24 h potentiated the activation of *PR1* gene expression upon bacterial inoculation, in addition to the direct activation of defence‐related genes through the transcription factor ARR2. In a separate study, pretreatment with the artificial CK 6‐benzylaminopurine (BAP) was shown to enhance resistance in *Arabidopsis* against the virulent oomycete *Hyaloperonospora arabidopsidis* (Hpa) isolate Noco2 in a dose‐dependent manner. While SA‐responsive defence genes were only slightly upregulated in response to BAP pretreatment, inoculation with Hpa Noco2 further enhanced the expression of these genes in comparison with dimethyl sulphoxide (DMSO)‐treated plants (Argueso et al. 2012). Notably, in both studies, CK pretreatment did not enhance pathogen resistance in the SA signalling mutant *npr1* nor in the SA‐deficient mutant *eds16*, indicating a crucial role of the SA pathway in CK‐mediated priming and/or resistance (Choi et al. 2010; Argueso et al. 2012). Consistent with these findings in *Arabidopsis*, studies of IR in tomato demonstrated that CK does not increase the resistance to Pst infection in an SA‐deficient background (Gupta et al. 2021). Additionally, feeding tobacco leaves with various CKs before bacterial infection significantly enhanced resistance to the hemibiotrophic pathogen *P. syringae* pv. *tabaci*. In this case, the CK effect was strongly correlated with increased levels of two major antimicrobial phytoalexins, scopoletin and capsidiol. These phytoalexins could substitute the CK signal to increase resistance against Pst infection, proving their functional relevance and suggesting a different mechanism for CK action in tobacco than observed in *Arabidopsis* (Großkinsky et al. 2011). More recently, CK‐arabinosides (CK‐A), which are CK sugar conjugates, were reported to enhance resistance to biotic stress (Bryksová et al. 2020). Foliar application of CK‐A to field‐grown wheat and barley plants suppressed infection by a fungal pathogen. From the analysis of the transcriptomic response of detached leaves of *Arabidopsis* to CK‐A treatment by RNA‐seq it was concluded that CK‐A may operate through activation of MAPK signalling cascades, in particular the PTI response. Notably, expression of *MPK11*, which is involved in PTI signalling, was strongly upregulated upon CK‐A treatment. The jasmonic acid (JA)/ethylene (ET) signalling pathway was enhanced by CK‐A treatment as indicated by upregulation of several defence genes, including *PLANT DEFENSIN 1.2* (*PDF1.2*) and increased levels of JA. In addition, the levels of reactive oxygen species (ROS) were temporarily elevated. Co‐treatment of CK‐A with the elicitor peptide flg22 showed strong synergy and increased expression of *FRK1*, a downstream marker of the MAPK defence pathway. The transcriptional response did not show any significant upregulation of CK response genes, suggesting that CK‐A activity is unlikely to be mediated by the canonical CK signalling pathway through the two‐component system (Bryksová et al. 2020).

It has been suggested that CK‐A enhances pathogen resistance through chemical priming (Bryksová et al. 2020). However, experiments demonstrating the effect of CK‐A pretreatment on pathogen infection in *Arabidopsis* under controlled conditions are lacking, as Bryksová et al. (2020) primarily focused on leaf longevity rather than resistance to biotic stresses. We were therefore interested in investigating chemically induced resistance by 6‐(3‐methoxybenzylamino)purine‐9‐arabinoside (BAPA) in more detail and chose Pst infection in *Arabidopsis thaliana* under in vivo conditions as an experimental system. The primary objective was to compare the ability of the CK conjugate BAPA to enhance bacterial resistance with that of the canonical CK, *t*Z. In addition to wild‐type (WT) plants, we tested the resistance‐inducing capacity of *t*Z and BAPA in a series of CK and stress hormone signalling mutants. Stress‐related parameters and transcriptomic responses were analysed to identify similarities and differences between the effects of *t*Z and the CK sugar conjugate BAPA. The results confirm the ability of both *t*Z and BAPA to induce resistance and highlight their common dependence on the CK receptor AHK3 and the SA signalling regulator NPR1. However, the findings also reveal differences in the action of the two compounds, as evidenced by their unique but also partially overlapping gene activation profiles.

## 2 Results

### 2.1 Inducing Resistance to Bacterial Infection

**FIGURE 1:**
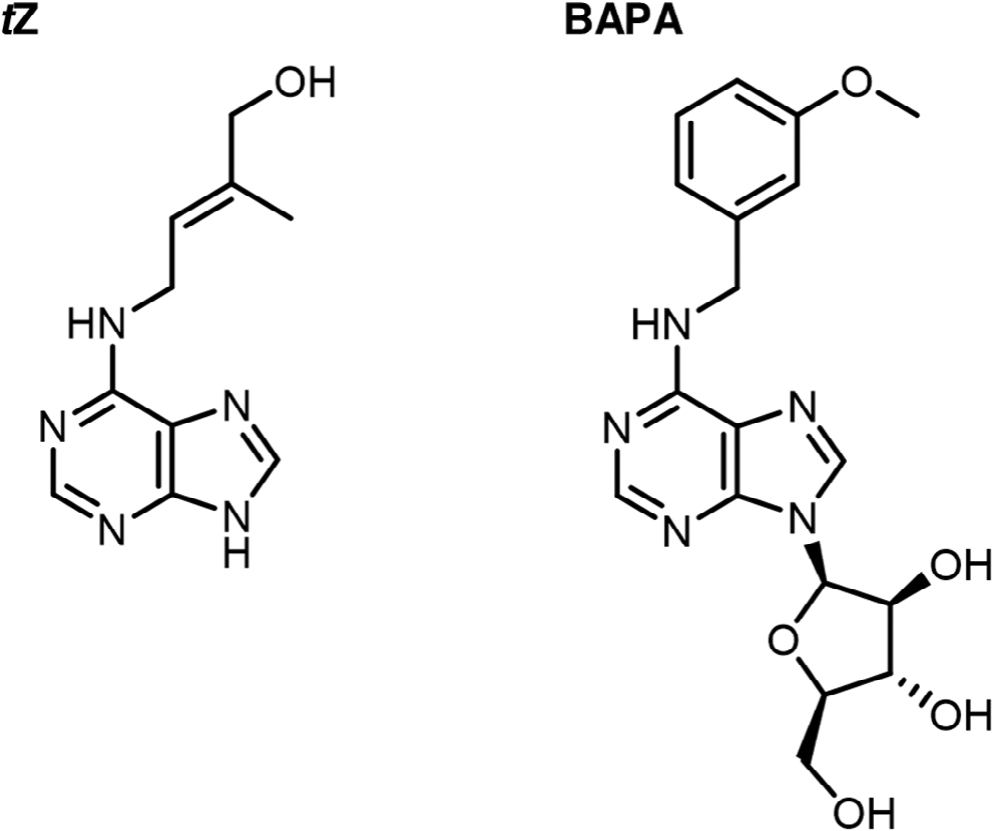
Chemical structure of *trans*‐zeatin (tZ) and 6‐(3‐methoxybenzylamino)purine‐9‐arabinoside (BAPA).

**FIGURE 2:**
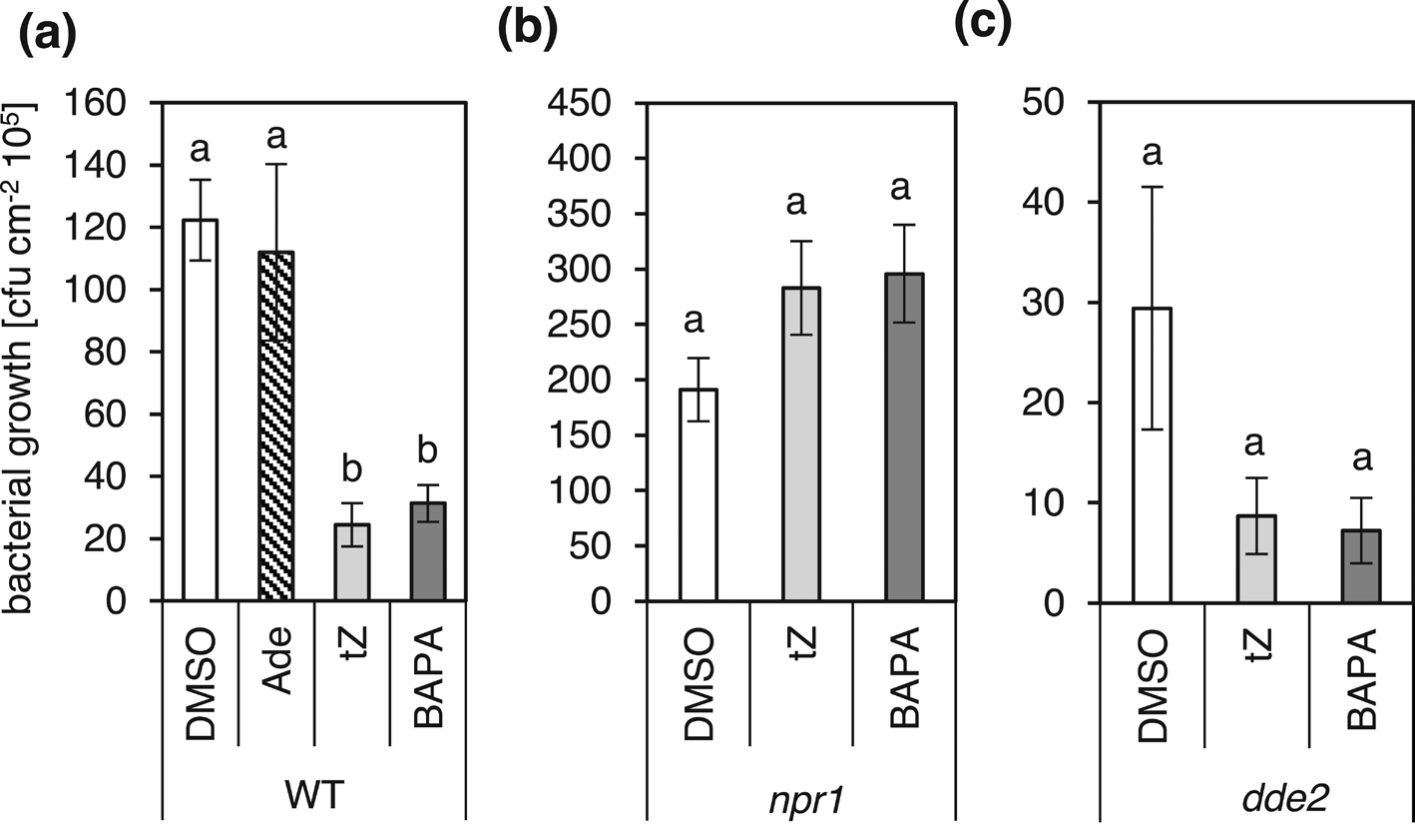
Chemically induced resistance of *Arabidopsis* against infection by *Pseudomonas syringae* . Bacterial growth 3 days post‐infection after treatment of *Arabidopsis* wild type (WT) (a) or the hormone mutants *npr1* (b) and *dde2* (c) with diemthyl sulphoxide (DMSO, solvent control), adenine (Ade, negative control), 6‐(3‐methoxybenzylamino)purine‐9‐arabinoside (BAPA) or *trans*‐zeatin (*t*Z). Compounds were sprayed on 4‐week‐old plants 1 day before the infection. Bars indicate the mean ± SE of colony‐forming units (cfu) per square centimetre from eight biological replicates, each consisting of four leaf discs from two leaves. Statistically different groups are indicated by different letters (*p* ≤ 0.05, Kruskal–Wallis test and post hoc Dunn's test with Benjamini–Hochberg *p*‐value correction method).

The effects of BAPA and *t*Z (Figure 1) on the defence response were evaluated following infection with Pst of 4‐week‐old soil‐grown *A. thaliana* plants (Figure 2). 0.05% DMSO was used as a solvent control; it showed a similar result to treatment with water. Adenine served as a negative control and exhibited no activity in this assay. In contrast, treatment with 50 μM BAPA induced a strong defence response, and bacterial growth was reduced to about 20% of the control treatment. A similarly increased defence response was caused by treatment with 1 μM *t*Z (Figure 2a).

A possible direct toxicity of BAPA or *t*Z on Pst was tested via growth curve analysis (Figure S1). Bacteria were grown in media supplemented with 0.1 μM to 50 μM BAPA or 0.1 μM to 10 μM *t*Z. This test did not reveal any influence of these compounds on the growth of Pst. In contrast, 10 or 50 μM H_2_O_2_ caused a significant inhibition of the growth of Pst. These data suggest that the observed biological effect (Figure 2) is likely caused by the ability of BAPA and *t*Z to enhance the defence capacity of treated plants rather than by a direct toxic impact of the compounds on Pst.

Next, we tested the impact of chemical treatment on the defence response of a mutant of the SA signalling pathway, *npr1* and of the JA biosynthesis mutant *dde2* (Figure 2b). *npr1* did not respond to either BAPA or *t*Z treatment. This is consistent with a previous study demonstrating the critical role of NPR1 in CK‐mediated defence against Pst infection in *Arabidopsis* (Choi et al. 2010). However, both BAPA and *t*Z were able to induce resistance in *dde2* to a similar degree as in WT (Figure 2c), indicating that JA does not play a role in resistance induced by BAPA or *t*Z or in defence against Pst.

**FIGURE 3:**
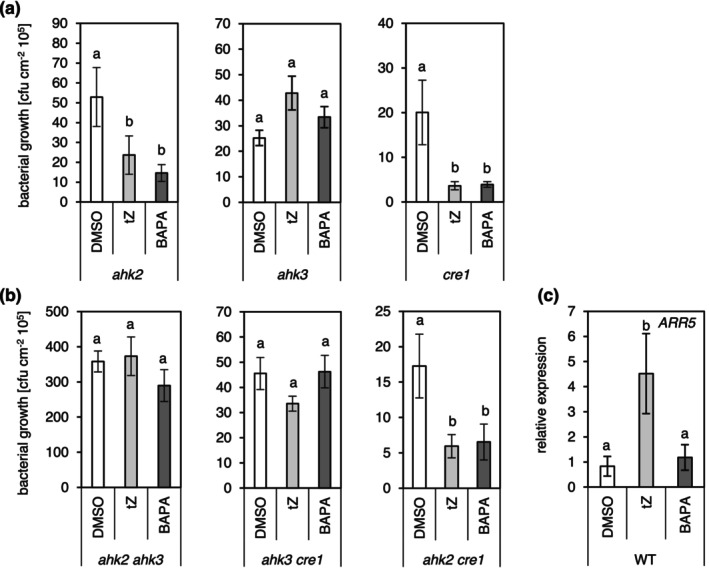
Chemically induced resistance of *Arabidopsis* against infection by *Pseudomonas syringae* . Bacterial growth 3 days post‐infection after treatment of the cytokinin (CK) receptor single (a) and double (b) mutants with dimethyl sulphoxide (DMSO, solvent control), 6‐(3‐methyoxybenzylamino)purine‐9‐arabinoside (BAPA) or *trans*‐zeatin (*t*Z). Compounds were sprayed on 4‐week‐old *Arabidopsis* plants 1 day before the infection. WT, wild type. Bars indicate the mean ± SE of colony‐forming units (cfu) per square centimetre from eight biological replicates, each consisting of four leaf discs from two leaves. Statistically different groups are indicated by different letters (*p* ≤ 0.05, Kruskal–Wallis test and post hoc Dunn's test with Benjamini–Hochberg *p*‐value correction method). (c) Expression of the CK reporter gene *ARR5* as measured by reverse transcription‐quantitative PCR 24 h after treatment with BAPA or *t*Z. Bars indicate the mean ± SD (*n* = 4). Different letters denote significant differences at *p* ≤ 0.05 (one‐way ANOVA and post hoc Tukey's HSD test).

To find out whether the ability of *t*Z and BAPA to induce resistance to bacterial infection depends on distinct CK receptors or their combinations, the activity of these compounds was tested in three single CK receptor mutants (*ahk2*, *ahk3* and *cre1*) and the three corresponding double mutants (*ahk2ahk3*, *cre1ahk2* and *cre1ahk3*). Of the single mutants, only *ahk3* showed no response to chemical treatment and did not exhibit a reduction in bacterial growth (Figure 3a), while *ahk2* and *cre1* responded similarly to WT (see Figure 2a). Among the double mutants, *cre1ahk2* was the only one to show a reduction in bacterial growth following treatment with *t*Z or BAPA, while this response was absent in *ahk2ahk3* and *cre1ahk3* (Figure 3b). This result indicates a critical role of the AHK3 receptor in mediating chemically induced resistance by both compounds. Notably, BAPA and *t*Z showed a similar activity to induce resistance to bacterial infection in all tested mutants. Further comparison of the two compounds revealed that treatment with BAPA did not induce the expression of the CK response gene *ARR5*, whereas treatment with *t*Z strongly induced it (Figure 3c). This surprising result indicates that although chemically triggered IR by BAPA depends on the receptor AHK3, as does IR by *t*Z, yet another typical CK activity, *ARR5* gene induction, is lacking. This is consistent with the study by Bryksová et al. (2020), which reported only negligible changes in *ARR* gene expression in response to different CK arabinosides.

### 2.2 Affinity to the AHK3 Receptor

**FIGURE 4:**
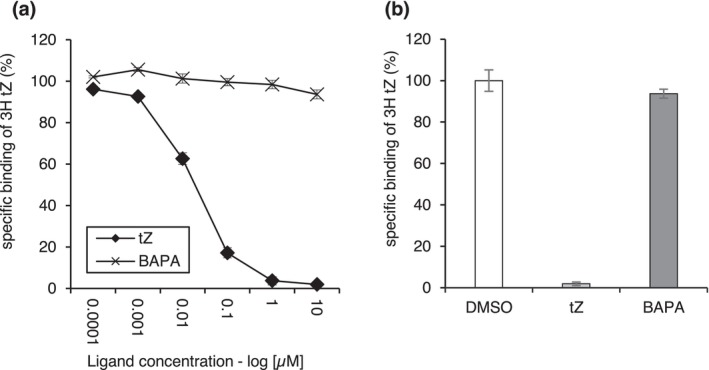
Competition assay of *trans*‐zeatin (*t*Z) and 6‐(3‐methoxybenzylamino)purine‐9‐arabinoside (BAPA) with [^3^H]‐*t*Z for binding to the AHK3 receptor heterologously expressed in *Escherichia coli* . (a) The specific binding of [^3^H]‐*t*Z in the presence of competitors, tested over a concentration range of 0.0001 to 10 μM, is expressed as a percentage relative to the dimethyl sulphoxide (DMSO) control. (b) The binding of [^3^H]‐*t*Z in the presence of competitors at a concentration of 10 μM, expressed as a percentage of the DMSO control, was: *tZ*—1.9%, BAPA—93.7%. Bars indicate the mean ± SD (*n* = 3).

Intriguingly, both tested compounds required AHK3 for the induction of resistance, but only *t*Z and not BAPA induced a typical CK‐dependent downstream response, like the induction of *ARR5*. BAPA is a sugar conjugate of 3‐methoxy‐BAP, which is a highly active CK (Figure 1). Typically, CK sugar conjugates display low or no CK activity and also show low or no affinity to CK receptors (Spíchal et al. 2004; Romanov et al. 2006; Lomin et al. 2015). Therefore, we analysed the affinities of *t*Z and BAPA to AHK3 in a bacterial assay. As was reported previously (Romanov et al. 2006), *t*Z binds to AHK3 with high affinity. In contrast, BAPA was not recognised by AHK3 in the competition assay (Figure 4).

### 2.3 Transcriptomic Changes in Response to Chemical Treatment

The previous result had shown a similar protective activity of *t*Z and BAPA but a difference in their ability to induce expression of *ARR5*, indicating a differential response of *Arabidopsis* to the two compounds. To investigate this further, we compared genome‐wide changes in gene expression caused by foliar application of *t*Z and BAPA. To this end, pairwise comparisons were made between each compound and DMSO treatment. Leaf samples were collected 24 h post‐treatment, aligning with the timing of bacterial inoculation in the infection assay.

**FIGURE 5:**
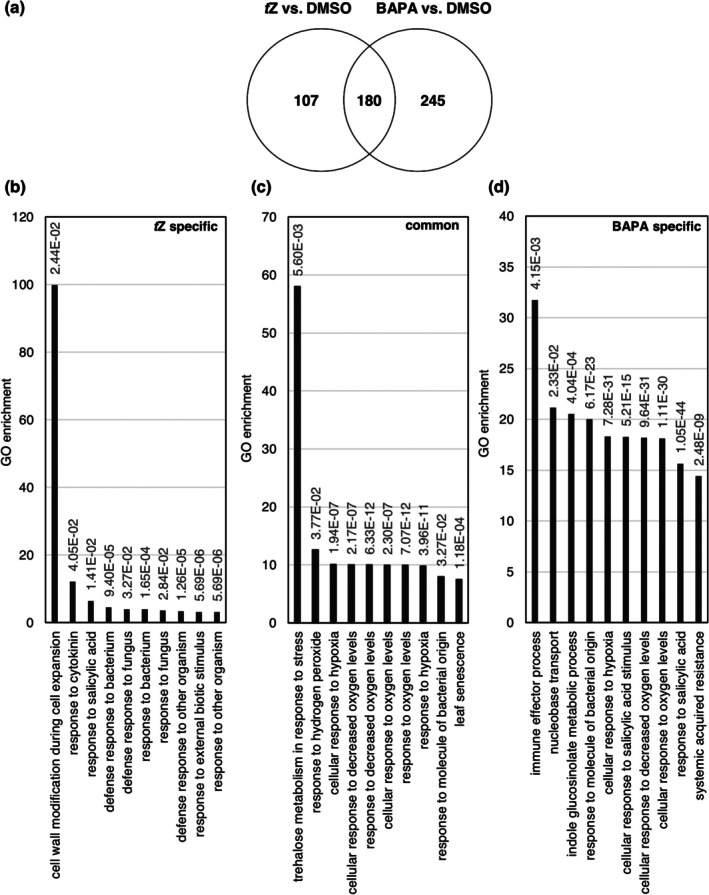
Transcriptomic changes in response to chemical treatment. Venn diagram (a) showing the numbers of differentially expressed genes (DEGs) and their overlap 24 h after 6‐(3‐methoxybenzylamino)purine‐9‐arabinoside (BAPA) or *trans*‐zeatin (*t*Z) treatment of *Arabidopsis* plants compared with dimethyl sulphoxide (DMSO)‐treated plants (log_2_ fold change ≥ 1; Benjamini–Hochberg‐corrected *p*‐value ≤ 0.05). Top 10 GO enrichment terms for *t*Z (b), common to *t*Z and BAPA (c) and BAPA (d) treatment. The complete list of DEGs used for (a) can be found in Table S2, and the result of the analysis shown in (a) is in Table S3.

A total of 463 differentially expressed genes (DEGs) were identified for BAPA treatment, compared to 322 for *t*Z (Table S2). Of these, a majority were upregulated: 425 DEGs (91.8%) following BAPA treatment and 287 DEGs (89.1%) after *t*Z treatment. Among these DEGs, 180 responded to treatments by both compounds, 245 genes responded specifically to BAPA, and 107 responded exclusively to *t*Z (Figure 5a, Table S3).

Only *t*Z treatment was found to broadly induce the expression of genes associated with CK responses (Table 1). Among the CK response genes, only *CYTOKININ OXIDASE 4* (*CKX4*), which encodes an enzyme catalysing the degradation of CKs, showed significantly increased expression after BAPA treatment with a log_2_ fold change ≥ 1 in comparison to DMSO control (Table 1). This result confirmed the lack of a transcriptomic CK‐like response to BAPA treatment.

**TABLE 1:** Selection of cytokinin response genes responding to chemical treatment.

Gene name	AGI	*t*Z (log_2_FC)	BAPA (log_2_FC)	*t*Z *p*_adj_	BAPA *p*_adj_
*ARR5*	AT3G48100	**2.09**	**0.75**	1.4E‐34	0.00020
*ARR6*	AT5G62920	**1.44**	0.20	3.1E‐13	0.57465
*ARR7*	AT1G19050	**1.03**	0.31	2.0E‐09	0.18769
*ARR15*	AT1G74890	**1.19**	−0.62	0.00136	0.18755
*CKX4*	AT4G29740	**1.28**	**1.13**	4.5E‐06	0.00006

*Note:* Only genes with a log_2_FC ≥ 1 are listed. Statistically significantly regulated genes are shown in bold (Benjamini–Hochberg‐corrected *p*‐value ≤ 0.05). BAPA, 6‐(3‐methoxybenzylamino)purine‐9‐arabinoside; FC, fold change; *p*_adj_, adjusted *p*‐value; *t*Z, *trans*‐zeatin. The complete dataset is shown in Table S4.

The treatment‐dependent DEGs with a log_2_ fold change ≥ 1 (Benjamini–Hochberg‐corrected *p*‐value ≤ 0.05) were further analysed by gene ontology (GO) term over‐representation analysis (Figure 5b). The most strongly enriched terms in response to *t*Z treatment were ‘cell wall modification during cell wall expansion’ and ‘response to cytokinin’. Interestingly, the next eight most enriched terms 24 h after *t*Z treatment dealt with the response to biotic stress, including the responses to bacteria and fungi as well as to SA. The enriched GO terms in response to BAPA included ‘immune effector processes’, ‘cellular responses to SA’ and ‘systemic acquired resistance’. Notably, both BAPA and *t*Z treatments influenced distinct sets of DEGs responsive to SA (Table 2, Table S4).

**TABLE 2:** Selection of salicylic acid signalling‐related genes responsive to chemical treatment.

Gene name	AGI	*t*Z (log_2_ FC)	BAPA (log_2_ FC)	*t*Z *p*_adj_	BAPA *p*_adj_
*ALD1*	AT2G13810	**2.25**	0.99	0.02536	0.49542
*ANAC055*	AT3G15500	**3.64**	**3.53**	0.01073	0.01161
*ANAC072*	AT4G27410	0.74	**1.91**	0.37599	0.00066
*CBP60g*	AT5G26920	0.78	**1.70**	0.17810	0.00009
*NPR3*	AT5G45110	**0.79**	**1.45**	0.00341	2.3E‐10
*SARD1*	AT1G73805	1.12	**1.69**	0.16191	0.01015
*WRKY46*	AT2G46400	0.62	**1.22**	0.18538	0.00066
*WRKY48*	AT5G49520	**1.07**	0.79	0.02516	0.13525
*WRKY70*	AT3G56400	0.56	**1.26**	0.52124	0.03620

*Note:* Only genes with log_2_ FC ≥ 1 are listed. Statistically significantly regulated genes are shown in bold (Benjamini‐Hochberg‐corrected *p*‐value ≤ 0.05). BAPA, 6‐(3‐methoxybenzylamino)purine‐9‐arabinoside; FC, fold change; *p*_adj_, adjusted *p*‐value; *t*Z, *trans*‐zeatin. The complete dataset is shown in Table S4.

Additionally, both treatments shared DEGs related to stress responses, including ‘response to hydrogen peroxide’ and ‘response to hypoxia and oxygen’. Both BAPA and *t*Z also activated genes associated with responses to molecules of bacterial origin. However, BAPA treatment uniquely upregulated additional specific DEGs related to this GO term, including *CBP60G*, *SARD1*, *WRKY40*, ABC transporter G family member 36 (*ABCG36*) and *Hyper‐Sensitivity‐Related 4* (*HSR4*) (Figure 5b, Table S2).

A more detailed analysis of genes associated with the biological processes identified through GO term analysis, particularly those that were strongly expressed, revealed overlapping DEGs in response to BAPA and *t*Z treatment. However, as highlighted in the GO term analysis, both compounds additionally triggered the expression of specific DEGs. Expression of selected marker genes related to plant stress and defence signalling is summarised in Table 3, Table S4. Genes specifically induced by BAPA include WRKY transcription factor genes (e.g., *WRKY33*, *WRKY40*), as well as SA (e.g., *CBP60G*, *SARD1*) and ethylene (ET) (*ERF6*) signalling markers. Moreover, genes responsible for pattern‐triggered immunity (PTI) signal amplification (e.g., *PREPIP2*) and pathogen‐associated molecular pattern (PAMP) signalling (e.g., *PH1*, *IDL6*) were expressed at significantly higher levels after BAPA treatment than after *t*Z treatment.

**TABLE 3:** Selection of strongly differentially expressed genes belonging to indicated categories 24 h after *trans*‐zeatin (*t*Z) or 6‐(3‐methoxybenzylamino)purine‐9‐arabinoside (BAPA) application.

Category	Gene name	ATG number	*t*Z (log_2_FC)	BAPA (log_2_FC)	*t*Z *p*_adj_	BAPA *p*_adj_
Stress response	*DMR6*	AT5G24530	**1.49**	0.95	0.026	0.220
	*MKK9*	AT1G73500	0.56	**1.56**	0.170	7.5e−8
	*MLO12*	AT2G39200	**1.78**	**1.71**	5.0e−5	9.0e−5
	*PDF1.2b*	AT2G26020	**3.82**	**2.42**	3.1e−6	0.008
	*PLA2A*	AT2G26560	**1.51**	**2.05**	0.027	0.007.3e−4
	*WAKL10*	AT1G79680	1.15	**2.22**	0.276	0.004
	*WRKY15*	AT2G23320	0.76	**1.55**	0.053	9.0e−7
	*WRKY22*	AT4G01250	0.56	**1.45**	0.386	7.3−4
	*WRKY33*	AT2G38470	1.06	**2.64**	0.216	2.0e−5
	*WRKY40*	AT1G80840	0.81	**2.26**	0.496	0.003
Salicylic acid response	*ALD1*	AT2G13810	**2.25**	0.99	0.02536	0.495
	*ANAC055*	AT3G15500	**3.64**	**3.53**	0.011	0.012
	*ANAC072*	AT4G27410	0.74	**1.91**	0.376	6.6e−4
	*CBP60g*	AT5G26920	0.78	**1.70**	0.178	9.0e−5
	*NPR3*	AT5G45110	**0.79**	**1.45**	0.003	2.3e−10
	*SARD1*	AT1G73805	1.12	**1.69**	0.162	0.010
ROS‐related	*GRXS13*	AT1G03850	**2.33**	**2.51**	1.2e−9	9.2e−12
	*MDAR3*	AT3G09940	**1.50**	**1.19**	0.003	0.028
	*PER33*	AT3G49110	**2.12**	1.32	0.04648	0.38913
	*PRX4*	AT1G14540	2.34	**4.32**	0.116	1.5e−4
Ethylene response	*ERF104*	AT5G61600	0.82	**2.33**	0.513	0.004
	*ERF5*	AT5G47230	0.79	**2.64**	0.407	1.0e−5
	*ERF6*	AT4G17490	1.21	**3.27**	0.3144	6.0e−5
	*PDF1.2a*	AT5G44420	**2.18**	1.44	0.0103	0.132
	*RAV2*	AT1G68840	0.92	**1.64**	0.0421	1.0e−5
PAMP	*IDL6*	AT5G05300	1.65	**2.35**	0.087	0.004
	*PH1*	AT4G14450	1.02	**3.03**	0.306	9.9e−07
	*PREPIP1*	AT4G28460	4.36	**6.65**	0.060	2.4e−4
	*PREPIP2*	AT4G37290	1.41	**3.34**	0.142	7.2e−7
	*PUB22*	AT3G52450	0.85	**1.49**	0.278	0.013

*Note:* Fold changes are compared to the dimethyl sulphoxide (DMSO) control. Statistically significantly regulated genes are shown in bold (Benjamini–Hochberg‐corrected *p*‐value ≤ 0.05). FC, fold change; *p*_adj_, adjusted *p*‐value. A more comprehensive analysis of cytokinin (CK)‐, salicylic acid (SA)‐, jasmonic acid (JA)‐ and reactive oxygen species (ROS)‐related genes is presented in Table S4. PAMP, pathogen‐associated molecular pattern.

**FIGURE 6:**
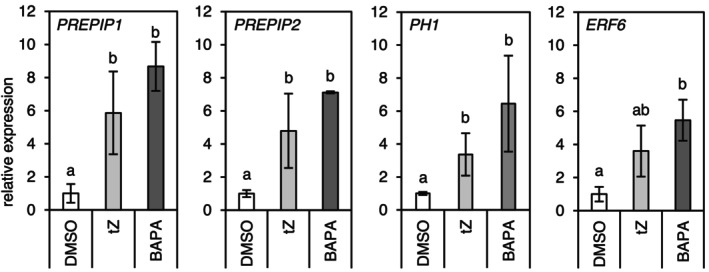
Relative gene expression measured by reverse transcription‐quantitative PCR. Relative expression of selected genes 24 h after treatment of 4‐week‐old wild‐type plants by *trans*‐zeatin (*t*Z) or 6‐(3‐methoxybenzylamino)purine‐9‐arabinoside (BAPA) (dimethyl sulphoxide [DMSO] control was set to 1). Bars indicate the mean ± SD (*n* = 4). Statistically different groups are indicated by different letters (*p* ≤ 0.05, one‐way ANOVA and post hoc Tukey's HSD test).

Selected genes were analysed using reverse transcription‐quantitative PCR (RT‐qPCR) (Figure 6). Genes associated with PAMP signalling (*PREPIP1*, *PREPIP2*, *PH1*) showed significantly increased expression 24 h after *t*Z or BAPA application; however, there was a stronger induction by the latter compound. This is consistent with the transcriptomic analysis, although the changes were not statistically significant for *t*Z (Table 3). Similarly, the RT‐qPCR analysis confirmed a strong induction of *ERF6* after BAPA treatment and a weaker induction by *t*Z.

### 2.4 Involvement of ROS

**FIGURE 7:**
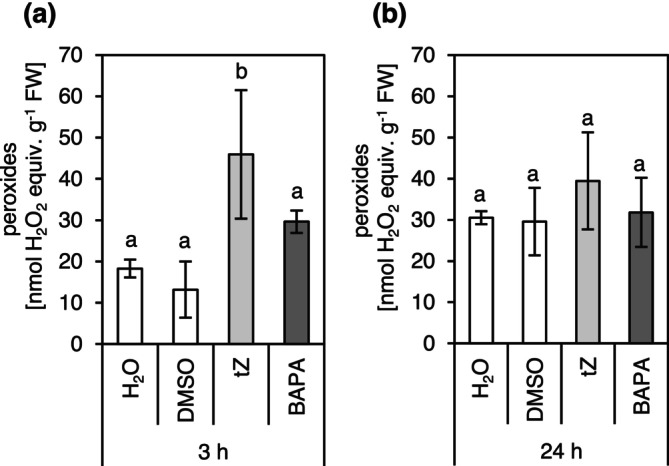
Concentration of peroxides after chemical treatment. *t*Z, *trans*‐zeatin; BAPA, 6‐(3‐methoxybenzylamino)purine‐9‐arabinoside. Peroxides were measured 3 h (a) and 24 h (b) after the indicated treatment of 4‐week‐old plants. Water and dimethyl sulphoxide (DMSO) are the corresponding controls. Bars indicate the mean ± SD (*n* = 4). Statistically different groups are indicated by different letters (*p* ≤ 0.05, one‐way ANOVA and post hoc Tukey's HSD test).

ROS play a crucial role in numerous stress responses. The oxidative burst represents the first line of defence against microbial invaders (Daudi et al. 2012; Dubiella et al. 2013). Transcriptomic data (Table 3) and RT‐qPCR results (Figure 6) showed induction of PAMP response genes after chemical treatment. To determine whether the treatments with the studied compounds might trigger an associated oxidative burst‐like response, peroxide concentrations were measured following chemical treatment. All tested compounds induced only mild increases in ROS as quantified by a peroxidase assay (Figure 7). An increase in ROS concentration 3 h after *t*Z treatment was statistically significant, but it returned to control levels by the time of bacterial inoculation (24 h after treatment). Overall, the data suggest that chemical treatment with the studied compounds results in only small changes of oxidative stress in the treated plants, at least at the tested time points, lending no support for a strong role for ROS in inducing plant resistance after *t*Z or BAPA treatment.

### 2.5 Effect on Plant Hormone Concentrations

**FIGURE 8:**
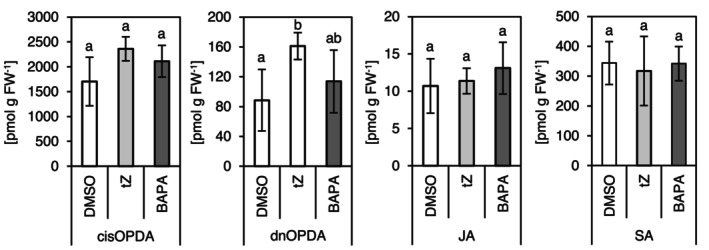
Concentrations of stress hormones. The hormone concentrations were measured in leaves number 8–10 of 4‐week‐old plants, 24 h after treatment with *t*Z or BAPA (DMSO is the solvent control). Bars indicate the mean ± SD (*n* = 4). Statistically different groups are indicated by different letters (*p* ≤ 0.05, one‐way ANOVA and post hoc Tukey's HSD test). BAPA, 6‐(3‐methoxybenzylamino)purine‐9‐arabinoside; cisOPDA, cis‐12‐oxo‐phytodienoic acid; dnOPDA, dinor‐12‐oxo‐phytodienoic acid; DMSO, dimethyl sulphoxide; JA, jasmonic acid; SA, salicylic acid; *t*Z, *trans*‐zeatin.

Next, we analysed whether the enhanced resistance to bacterial infection following treatment with BAPA or *t*Z was accompanied by an increase in stress hormone concentration. At first, as expected after *t*Z application, a strong increase of its concentration was observed, along with increased levels of the metabolites *t*Z riboside (*t*ZR) and *t*Z glucosides, indicating successful uptake through the leaves. In contrast, treatment by BAPA did not cause major changes in the endogenous CK concentration (Table S5). The concentration of the JA precursor dinor‐12‐oxo‐phytodienoic acid (dnOPDA) increased significantly following *t*Z application; however, JA levels remained unchanged (Figure 8; Table S5). Furthermore, the concentration of the highly active JA conjugate JA‐isoleucine (JA‐Ile) remained below the detection limit after both *t*Z and BAPA treatments. The concentration of SA was not significantly altered by any of the treatments 24 h after the treatments (Figure 8).

## 3 Discussion

The comparison of the ability of the canonical CK *t*Z and the CK sugar conjugate BAPA to induce resistance to pathogen infection in *Arabidopsis* has revealed both shared and unique features of their action. A significant protective effect was demonstrated by both substances, reducing bacterial growth to approximately 15%–20% of that in the negative controls in infected leaves. This confirmed earlier reports on the ability of various CKs (Argueso et al. 2012; Choi et al. 2010; Großkinsky et al. 2011) and of BAPA (Bryksová et al. 2020) to induce resistance to pathogen infection.

A functional SA signalling pathway was essential for the protective effect of both compounds. This is strongly supported by the requirement of NPR1 (Figure 2) and aligns with CK's known role in modulating SA signalling to augment resistance against Pst (Choi et al. 2010). Despite no significant increase in SA levels 24 h post‐treatment (Figure 6), transcriptomic data showed enrichment of SA‐responsive genes (Figure 3), suggesting enhanced SA sensitivity rather than increased SA biosynthesis being causative for enhanced resistance. The absence of major shifts in signalling molecule concentrations suggests that *t*Z and BAPA do not directly induce full defence responses but possibly enhance the plant's readiness to respond to pathogen attack (Hilker and Schmülling 2019; Mauch‐Mani et al. 2017). Noteworthy, CK has been shown to have priming activity (Argueso et al. 2012; Choi et al. 2010; Großkinsky et al. 2011), while direct evidence for BAPA is still missing.

The lack of enhanced bacterial resistance in the JA synthesis mutant *dde2*, and the similarity of the defence response in *dde2* and WT, ruled out the participation of the JA signalling pathway in increasing resistance to bacterial infection (Figure 2). Consistently, there was no enrichment of JA‐related genes among the DEGs induced by *t*Z and BAPA, and no increase in JA metabolite concentrations upon chemical treatment. This contrasts with Bryksová et al. (2020), likely due to methodological differences such as the use of detached leaves, which may cause wounding‐induced JA production (Kimberlin et al. 2022). However, both this study and the study of Bryksová et al. (2020) pointed to possible activation of the ET pathway as several ET signalling markers were induced by BAPA.

A third factor with a putative role in IR that was analysed is ROS, which are known to be involved in the defence response to biotic stress (Hilker et al. 2016). Indeed, ‘response to hydrogen peroxide’ was the second most enriched GO term among the common DEGs induced by both *t*Z and BAPA (Figure 5b). The peroxide concentration was slightly increased 3 h after treatment and returned to the baseline at the time of infection (Figure 7). Bryksová et al. (2020) also reported that BAPA‐induced ROS production peaks 3 h after BAPA treatment in *Arabidopsis* cell cultures, albeit at a higher level. The kinetics of changes in ROS concentration suggest an early or limited role in chemically induced resistance. Further kinetic and genetic studies are needed to clarify the involvement of ROS in CK‐triggered IR.

The transcriptomic response of *Arabidopsis* to BAPA suggested that BAPA operates through activation of the PTI response, particularly through MAPK signalling cascades involving WRKY transcription factors (Bryksová et al. 2020). Consistently, in our study, several WRKY transcription factor genes (*WRKY15*, *WRKY22*, *WRKY33*, *WRKY40*) and the gene encoding mitogen‐activated protein kinase 9 (*MKK9*) were induced by BAPA treatment. In addition, genes responsible for PTI signal amplification (*PREPIP2*) and PAMP signalling (*PH1*, *IDL6*) were responsive. Other SA (e.g., *CBP60G*, *SARD1*) and ET (*ERF6*) signalling markers induced by BAPA are also involved in defence signalling downstream of plant immune receptors (Peng et al. 2018). None of the PTI‐related genes were significantly upregulated in the RNA‐seq analysis following *t*Z treatment (Table 3), but RT‐qPCR assays (Figure 4a) revealed that *t*Z also induced their expression, albeit to a lesser extent, at least at the tested time points. These transcriptomic changes support the hypothesis that the protective effect of BAPA and *t*Z is at least partially mediated by the PTI pathway. This suggests a possible crosstalk between CK signalling and MAPK pathways, supported by recent findings showing MPK3/6‐mediated phosphorylation of B‐type ARRs (Yan et al. 2021). Nonetheless, neither Bryksová et al. (2020) nor our study tested PTI mutants for gene expression or defence activation, and thus, the importance of PTI components in the mode of action of BAPA remains to be validated.

A novel result of this study is that induction of IR in *Arabidopsis* by both *tZ* and BAPA requires the AHK3 receptor, indicating that the canonical CK response pathway is involved for both compounds. However, while *t*Z binds to AHK3 with a high affinity, BAPA was not recognised by AHK3 in a bacterial assay (Figure 4). This is in line with the sole recognition of the free bases by CK receptors (Lomin et al. 2015; Romanov and Schmülling 2022). Therefore, the requirement of AHK3 for the activity of BAPA was surprising. The *AHK3* receptor gene is expressed broadly in leaf tissue (Higuchi et al. 2004), where it is active in parenchyma and stomatal cells (Stolz et al. 2011), which is consistent with a function in defence. An unusual finding is that AHK3 acts alone and not together with either AHK2 or CRE1/AHK4, which is most often the case (e.g., Cortleven et al. 2014; Kim et al. 2006; Riefler et al. 2006). The latter two receptors are not functionally relevant for IR, as *ahk2*, *cre1* and *cre1 ahk2* mutants show no altered response to bacterial infection (Figure 2). The ligand recognition spectrum of AHK3 is generally broader than that of the two other receptors, and it displays a significantly higher sensitivity towards *t*Z than towards another biologically active CK, isopentenyladenine (iP) (Spíchal et al. 2004; Romanov et al. 2006; Lomin et al. 2015). Furthermore, AHK3 is the main receptor mediating CK action on leaf senescence (Kim et al. 2006; Riefler et al. 2006). Notably, retardation of leaf senescence is an activity shared by BAPA and other structurally related CK N9‐conjugates (Hönig et al. 2018), although the latter are also only poorly recognised by CK receptors (Lomin et al. 2015; Romanov et al. 2006; Spíchal et al. 2004).

Despite the common requirement of AHK3, the DEGs induced by BAPA and *t*Z were only partially identical and BAPA did not induce primary CK response genes such as *ARR5*, which is consistent with Bryksová et al. (2020). The sole exception in this respect has been the induction of the *CKX4* gene, encoding a CK‐degrading CK oxidase/dehydrogenase enzyme and belonging to the most sensitive and commonly induced CK response genes (Brenner et al. 2012). Its activation by both BAPA and *t*Z indicates a homeostatic response to increased CK signalling caused by both compounds.

In addition to the lack of CK response gene induction, BAPA exhibited only low activity in several CK bioassays, except for high activity in delaying leaf senescence (Bryksová et al. 2020; Vylíčilová et al. 2020). This pattern of selective CK activity, including the lack of activation of CK response genes except *CKX4*, has also been observed with other CK sugar conjugates, which can present the majority of CK metabolites in the cell (Doležal et al. 2007; Hošek et al. 2020; Matušková et al. 2020; Pokorná et al. 2021; Vylíčilová et al. 2020; reviewed by Hluska et al. 2021; Hoyerová and Hošek 2020; Vylíčilová et al. 2020). This indicates that some CK derivatives, including BAPA, specifically influence physiological processes related to senescence and stress without being recognised by CK receptors and, therefore, without showing other typical CK activities.

How could BAPA activate IR through AHK3 without being recognised by the receptor? A slow release of free base from BAPA might be sufficient to support certain lasting CK activities, including causing IR and retarding senescence processes, but not to activate response gene expression. However, so far, investigation of the hydrolysis of N9‐glucosides has yielded conflicting results (Hallmark et al. 2020; Hošek et al. 2020; Podlešáková et al. 2012; Pokorná et al. 2021). The release of biologically active free base by glycosidases from BAPA has not yet been tested, but in view of the notoriously low substrate specificity of these enzymes, it is an option that needs to be explored. Yet another possibility is that AHK3 is required to maintain a basic background level of CK signalling from endogenous CK but does not transmit a BAPA signal. In any case, the involvement of AHK3 in BAPA action strongly suggests that the canonical CK signalling pathway is involved in its activities.

Taken together, genetic analysis and transcriptomic profiling have confirmed that SA signalling is important to induce enhanced resistance to Pst infection by CK. Transcriptomic analyses suggest that ROS and MAPK signalling are relevant downstream processes. In contrast, support for a role of JA has not been found. Importantly, the canonical CK signalling pathway needs to be activated for BAPA and *t*Z to be efficient in inducing pathogen resistance. However, there are differences in the downstream responses of these compounds, suggesting that specific stimuli induce partly overlapping but also distinct responses upon activation of the same CK receptor. How this is achieved mechanistically needs further studies.

Our results highlight BAPA as a promising candidate for plant immunity induction, potentially offering a novel approach for crop protection without major alterations to CK homeostasis. Given its ability to enhance resistance without drastic metabolic shifts, a stimulatory effect on the number of productive tillers and grain yield, BAPA or similar CK conjugates could be explored as derivatives of a naturally occurring compound for their usage to improve disease resistance of crop plants. Although our results significantly broaden the understanding of the protective effect of BAPA, demonstrating for the first time its ability to protect plants in vivo against bacterial pathogens, further research is required to fully characterise its IR phenotype and, in particular, its ability to prime the defence response. A further interesting question is whether these substances can contribute to uncoupling defence and growth trade‐offs, a role which has recently been reported for CK (Leibman‐Markus et al. 2023).

## 4 Experimental Procedures

### 4.1 Plant Material and Growth Conditions

*Arabidopsis thaliana* accession Col‐0 was used as the wild type (WT). The *npr1* (EMS‐mutant N3726) (Cao et al. 1997), the *dde2* (von Malek et al. 2002), and the cytokinin receptor mutants *ahk2 ahk3*, *ahk2 cre1* and *ahk3 cre1* (Riefler et al. 2006) have been published. *Arabidopsis* plants were grown on soil for 4 weeks under short day (SD) conditions (8 h light/16 h darkness) in cultivation shelves (Photon Systems Instruments) with light intensities of 100–150 μmol m^−2^ s^−1^, using light‐emitting diode (LED) lighting (cool white LEDs and far‐red LEDs), at 22°C and 60% relative humidity.

### 4.2 Chemical Treatment

BAPA was prepared according to Bryksová et al. (2020). Stock solutions of *t*Z (2 mM), BAPA (100 mM) and adenine (Ade) (100 mM) were prepared in DMSO. Just before foliar application, these solutions were diluted with water to achieve the desired concentrations of 50 μM for BAPA and Ade, and 1 μM for *t*Z. The final concentration of DMSO in the mixture was in all cases 0.05%. 20 mL of the final solution was uniformly sprayed onto the foliage of eight plants until all leaves were evenly covered with droplets.

### 4.3 Bacterial Growth Assessment

Three leaves (numbers 8–10) of 4‐week‐old plants (eight biological replicates per treatment) were syringe‐infiltrated from the abaxial side with Pst DC3000 (OD_600_ = 0.002 for bacterial growth assessment, 0.005 for gene expression analysis) 24 h after chemical treatment. Bacterial growth was assessed as described by Griebel and Zeier (2008) with some modifications. Luria Bertani (LB) medium containing rifampicin and cycloheximide (final concentrations 50 μg mL^−1^) was used for bacterial cultivation and plating. Bacteria were extracted from two leaves per plant by homogenisation in 0.1 mL of 10 mM MgCl_2_ 3 days after inoculation.

### 4.4 Cytokinin Binding Assay

The cytokinin binding assay was performed as a competition assay according to the protocol described by Romanov et al. (2006). Bacteria were cultured overnight in M9 medium supplemented with 25 μg mL^−1^ chloramphenicol and 0.1% (wt/vol) casamino acids at 25°C under continuous agitation (150 rpm) until an OD_600_ of approximately 0.8 was reached. Protein expression was induced by the addition of 0.5 mM isopropyl‐β‐D‐thiogalactopyranoside (IPTG), and cultures were incubated for an additional 6 h at 18°C, 150 rpm.

An aliquot of 1 mL of the *Escherichia coli* suspension was transferred to a microtube and mixed with 1 μL of *t*Z or BAPA solution at the appropriate concentration (final concentration = 0.0001–10 μM) in DMSO. Subsequently, 2 μL of 1.5 μM [^3^H]‐*t*Z was added to each aliquot (final concentration = 3 nM). As a negative control, 1 μL of DMSO was added instead of cytokinin. A mixture of [^3^H]‐*t*Z and a large excess of unlabelled *t*Z was used to distinguish specific from nonspecific binding.

Following incubation for 30 min at 4°C, samples were centrifuged at 6000 *g* for 6 min at 4°C. The supernatant was removed using a vacuum pump, and the pellet was thoroughly resuspended in 1 mL scintillation cocktail. Radioactivity was measured using an SL6500 scintillation counter (Beckman Coulter, Ramsey, MN, USA) for 10 min per sample. The experiment was performed with three independent replicates. Mean values and standard deviations were calculated using Excel (Microsoft).

*t*Z standard was obtained from Olchemim. Highly labelled *trans*‐[2‐^3^H]zeatin ([^3^H]‐*t*Z; radiochemical purity > 99%) was obtained from the Isotope Laboratory of the Institute of Experimental Botany (Prague, Czech Republic). The *E. coli* strain carrying the pSTV28 vector expressing AHK3 was kindly provided by Dr. D. Zalabák from the Laboratory of Growth Regulators, Palacký University & Institute of Experimental Botany of the Czech Academy of Sciences (Olomouc, Czech Republic).

### 4.5 Real‐Time RT‐qPCR

Three leaves (numbers 8–10) of 4‐week‐old plants were used for total RNA extraction. RNA was extracted from the samples frozen in liquid nitrogen and homogenised using an MM200 bead mill (Retsch). TRI reagent (Sigma Aldrich) was used according to the manufacturer's instructions. Isolated RNA was then treated with DNase I (Fermentas) to prevent genomic DNA contamination. RNA samples (1 μg) were reverse‐transcribed using SuperScript III Reverse Transcriptase (Invitrogen) according to the manufacturer's recommendations. cDNA samples were used as templates in real‐time PCRs containing SYBR Green I, Immolase (Bioline), and 300 nM of each primer (Table S1). The PCR was performed using a CFX96 Touch Real‐Time Detection System (Bio‐Rad) with the following cycling conditions: 15 min 95°C; 40 cycles of 15 s at 95°C and 15 s at 55°C, and 10 s at 72°C; followed by the generation of a dissociation curve to check for the specificity of the amplification. Gene expression data were normalised against two reference genes (*PP2A* [*At1g13320*), *TFIID* [*At3g13445*) and/or *ACT2* [*At3g18780*]) according to Vandesompele et al. (2002).

### 4.6 Analysis of Gene Expression by RNA‐Seq

Plant cDNA sequencing libraries were prepared using the Illumina TruSeq Stranded mRNA Sample Preparation Kit and IDT for Illumina TruSeq Unique Dual Index Kit (96 indexes, Illumina), following the manufacturer's instructions. A total of 2 μg of total RNA per sample was used, with three independent biological replicates per condition to generate cDNA libraries. The resulting libraries were validated using a High Sensitivity DNA Kit with a 2100 Bioanalyzer (Agilent Technologies). For accurate determination of library concentrations, the Kapa Library Quantification Kit (Kapa Biosystems) was employed. Subsequently, the libraries were pooled to achieve a final concentration of 14 pM for cluster generation and sequencing. Sequencing was performed on the NovaSeq 6000 Sequencing Platform (Illumina) using the NovaSeq 6000 S1 Reagent Kit. Quality control for the sequencing reads was performed using FASTQC v. 0.11.5 (Anders et al. 2015), and reads were mapped to the reference genome of *A. thaliana* v.25 obtained from Ensembl (Cunningham et al. 2015) using a TopHat v. 2.0.12 splice‐read mapper (Kim et al. 2013) with default parameters. The reads mapped to the transcripts annotated in the reference genome were quantified using HTSeq v. 0.6.0 (Anders et al. 2015) with respect to the stranded library. RNA‐seq data are deposited in NCBI's BioProject database and are accessible through the accession number PRJNA1280743. Differential gene expression analysis was conducted using the DESeq2 method (Love et al. 2014) in R (v. 4.3.1) and RStudio. DEGs were identified based on a Benjamini–Hochberg‐corrected *p*‐value of ≤ 0.05 and log_2_ fold change values of ≥ 1. GO term enrichment analysis for the biological process aspect was performed using PANTHER (Mi et al. 2019) accessed via https://www.arabidopsis.org/tools/go_term_enrichment.jsp. CK‐, SA‐, JA‐ and ROS‐related gene lists used for the analysis in Table S4 were published by Cortleven et al. (2022). The lists of stress‐ and ET‐related genes used in Table 3 were adapted from Bhardwaj et al. (2011) and Rehrig et al. (2014), respectively.

### 4.7 Quantification of Plant Hormones

Three leaves (numbers 8–10) of 4‐week‐old plants treated with *t*Z, BAPA or DMSO (four biological replicates per treatment) were harvested 24 h after chemical treatment and immediately frozen in liquid nitrogen. Samples were homogenised using an MM400 bead mill (Retsch) at 27 Hz for 5 min, and 15 mg of the powdered sample was weighed into a microtube. The samples were covered with 1 mL of 60% (vol/vol) aqueous acetonitrile, and a mixture of stable isotope‐labelled internal standards was added in the concentrations published in Šimura et al. (2018). The samples were sonicated for 3 min and incubated for 30 min at 4°C with slow shaking. After the incubation, the microtubes were centrifuged for 15 min at 36,670 *g* and 4°C in an Allegra 64R benchtop centrifuge (Beckman Coulter). The collected supernatant was purified with Oasis HLB 30 mg/1 cc extraction cartridge (Waters). Briefly, the cartridge was activated with 1 mL of methanol and equilibrated with 2 mL of 60% (vol/vol) aqueous acetonitrile. The sample was loaded onto the cartridge and washed with 0.5 mL of 60% (vol/vol) aqueous acetonitrile and 0.5 mL of 30% (vol/vol) aqueous acetonitrile. Both the loading and wash fractions were collected in a 5 mL borosilicate glass tube (Thermo Fisher Scientific), and the purified sample was evaporated in a SpeedVac concentrator RC1010 Centrivap Jouan (Thermo Fisher Scientific). The dry sample was reconstituted in 40 μL of 25% (vol/vol) aqueous acetonitrile and transferred to a liquid chromatography vial equipped with a glass insert (Chromservis Ltd.). 5 μL was injected into an Acquity UPLC CSH C18 1.7 μm, 2.1 × 150 mm chromatography column (Waters) and analysed by ultrahigh performance chromatograph (Acquity UPLC I‐Class System; Waters) coupled to a Xevo TQ‐XS triple quadrupole mass spectrometer (Waters). The gradient elution and mass spectrometry conditions were set according to previously published methodology (Šimura et al. 2018). Data were acquired in multiple reaction monitoring mode. The chromatograms were evaluated using MassLynx v. 4.2 software (Waters), and the concentrations of plant hormones were determined by the isotope dilution method (Rittenberg and Foster 1940).

### 4.8 Determination of Peroxide Levels

Three leaves (numbers 8–10) of 4‐week‐old plants (approximately 100 mg) were flash‐frozen in liquid nitrogen and homogenised. Water‐soluble peroxides were quantified following the xylenol orange‐based method described in Abuelsoud et al. (2020), using the Pierce Quantitative Peroxide Assay Kit (Aqueous) (ThermoFischer Scientific). Hydrogen peroxide was used as the standard. The content of water‐soluble peroxides was expressed as nmol H_2_O_2_ equivalent g^−1^ FW.

## Author Contributions

Martin Hönig, Anne Cortleven and Thomas Schmülling developed and coordinated the project, designed experiments, and analysed data. Martin Hönig performed most experiments. Ivan Petřík measured plant hormone concentrations. Simerský Radim performed the cytokinin receptor affinity assay. Magdalena Bryksová prepared BAPA. Ondřej Plíhal performed RNA‐seq analyses. Martin Hönig and Thomas Schmülling wrote the article. All authors read and contributed to previous versions and approved the final version.

## Funding

This work was supported by Deutsche Forschungsgemeinschaft (DFG) in the frame of CRC 973 ‘Priming and memory of organismic responses to stress’ and the Federation of European Biochemical Societies (FEBS). Open Access Funding provided by Freie Universität Berlin.

## Conflicts of Interest

The authors declare no conflicts of interest.

## Supporting information


**Figure S1:** Test of cytotoxicity.

**Table S1:** Primers used for RT‐qPCR.

**Table S2:** Differentially expressed Arabidopsis genes 24 h after *t*Z or BAPA application.

**Table S3:** An overlap analysis of DEGs upregulated by *t*Z and BAPA treatments.

**Table S4:** Expression of CK/SA/JA/ROS‐related genes 24 h after *t*Z or BAPA application.

**Table S5:** Phytohormone concentrations 24 h after *t*Z or BAPA application.

## Data Availability

All data needed to evaluate the conclusions in the paper are present in the paper and the Supporting Information. The data that support the findings of this study are openly available in Zenodo at 10.5281/zenodo.16084024
