# Supplementary material for: Chemically Induced Resistance to Pathogen Infection in Arabidopsis by Cytokinin (Trans‐Zeatin) and an Aromatic Cytokinin Arabinoside
Source: Mol Plant Pathol. 2026 Jan 9;27(1):e70200. doi: 10.1111/mpp.70200 (PMC12789194; doi:10.1111/mpp.70200)
Supplement: Supplementary file 1 — Figure S1: Test of cytotoxicity. [file MPP-27-e70200-s005.docx]

SUPPORTING FIGURE 1 Test of cytotoxicity. (**A**) Exponential part of the bacterial growth curve after the application of studied compounds in various concentrations. H_2_O_2_ and DMSO are positive and negative controls. (**B**) Bacterial growth at selected time points of the exponential part of the bacterial growth curve. Bars indicate the mean ± SD of 6 technical replicates. Stars indicate statistically significant differences between indicated treatments (***P < 0.001, ANOVA).
