# Supplementary material for: Chemically Induced Resistance to Pathogen Infection in Arabidopsis by Cytokinin (Trans‐Zeatin) and an Aromatic Cytokinin Arabinoside
Source: Mol Plant Pathol. 2026 Jan 9;27(1):e70200. doi: 10.1111/mpp.70200 (PMC12789194; doi:10.1111/mpp.70200)
Supplement: Supplementary file 2 — Table S1: Primers used for RT‐qPCR. [file MPP-27-e70200-s006.docx]

**Supporting Table 1** Primers used for qRT-PCR.

| Gene | Forward primer sequence (5'-3') | Reverse primer sequence (3'-5') |
| --- | --- | --- |
| *ACT2* | CTTGCACCAAGCAGCATGAA | CCGATCCAGACACTGTACTTCCTT |
| *ARR5* | GCCGAAAGAATCAGGACA | CTACTCGCAGCTAAAACGC |
| *ERF6* | TCGAATCCTCCTCGCGTTACTG | TTCGGTGGTGCGATCTTCAACG |
| *FRK1* | GAAGCGGTCAGATTTCAACA | TCAAGAAGAACAACCCCAAGA |
| *PH1* | TGGCGTCCACGATGCAAAGAAC | CGTCTCTGTAGCTGGCTAAGTTGC |
| *PP2A* | CCATTAGATCTTGTCTCTCTGCT | GACAAAACCCGTACCGAG |
| *PR1* | ATGCAGTGGGACGAGAGGGT | AACCCACATGTTCACGGCGG |
| *PREPIP1* | AAGTGCGACTATGACGGTGGAG | GACGCCAAACGCTGAAACCAAG |
| *PREPIP2* | TGTTAAGCACTCAGGTCCAAGCC | CGGCCCGGTTTGAACTAAAGATTG |
| *TFIID* | ACTCTTAGCCAAGTAGTGCTCC | GAATCACGGCCAACAATC |
